# Factors influencing healthcare workers’ compliance with personal protective equipment guidelines in long-term care during the COVID-19 pandemic—A theory-based mixed-methods study

**DOI:** 10.1371/journal.pone.0321851

**Published:** 2025-04-29

**Authors:** Anna-Leena Lohiniva, Jaana Marija Lehtinen, Dinah Arifulla, Jukka Ollgren, Pekka Nuorti, Outi Lyytikäinen

**Affiliations:** 1 Finnish Institute for Health and Welfare, Helsinki, Finland; 2 University of Tampere, Faculty of Social Sciences, Health Sciences Unit, Tampere, Finland; University of Haifa, ISRAEL

## Abstract

**Introduction:**

Residents and healthcare workers in long-term care facilities are at increased risk of infection during respiratory epidemics. Proper compliance with infection prevention and control measures is therefore paramount. Our study aimed to uncover and understand factors influencing compliance with personal protective equipment guidelines among healthcare workers in long-term care facilities in Finland during the COVID-19 pandemic.

**Methodology:**

We conducted a mixed-methods study by using the methods and framework of behavioral insights, specifically the Theoretical Domains Framework. Data were collected through a web-based survey and qualitative in-depth telephone interviews using convenience sampling. Participants for the anonymous survey were recruited through regional infection control experts during May-June 2022. Survey data were analyzed by using logistic regression; difficulties in complying with personal protective equipment guidelines were the outcome. Volunteer survey respondents were interviewed, and the data were analyzed thematically.

**Results:**

A total of 373 healthcare workers participated in the survey; 56% had received personal protective equipment training. Two domains of the Theoretical Domains Framework were associated with experiencing difficulties in compliance with the personal protective barriers: organization linked with insufficient human resources and the presence of negative emotions linked with stress. Twenty-two healthcare workers participated in the interviews which resulted in the identification of several themes, suggesting how insufficient human resources and negative emotions affected personal protective equipment use and the type of coping mechanism that healthcare workers used to overcome these barriers.

**Conclusions:**

The behavioral insights derived from our study can contribute to enhanced healthcare worker compliance with personal protective equipment use. These findings underscore the importance of access to training, interventions addressing stress and ensuring sufficient workforce.

## Introduction

Long-term care facilities (LTCFs) experienced severe impacts during the global COVID-19 pandemic [1–4]. The residents of LTCFs, typically elderly individuals, often contend with underlying medical conditions that heighten their susceptibility to infections [5–7]. The elevated prevalence of functional impairment necessitates frequent close physical contact between residents and healthcare workers (HCWs). Moreover, a substantial number of LTCF residents grapple with underlying cognitive impairment or dementia, posing challenges for HCWs to adhere to infection prevention recommendations.

Infection prevention and control (IPC) guidelines play a pivotal role as they establish standards of practice, enabling the assessment of adherence and the evaluation of care quality. Amid the pandemic, guidelines for personal protective equipment (PPE) commonly encompassed the utilization of gloves, gowns, face shields or eye protection, surgical masks or FFP2/3 respirators [8,9]. In Finland, LTCFs received guidance and training on the proper use of PPE during COVID-19 patient care from multiple sources, including municipalities, healthcare districts, and national institutes [10].

The implementation of IPC practices in LTCFs faces challenges due to the diverse nature of these institutions, which may or may not provide skilled nursing care [11,12]. They include home care, sheltered housing with round-the-clock care services, nursing homes, and inpatient wards within healthcare centers Approximately 80% of the elderly in care reside in 24-hour service housing, offered by both public and private sectors. The average age of these residents is 84 years, and over half of them struggle with memory disorders [10].

The introduction of new IPC practices often necessitates alterations to established routine behaviors, a process that can prove challenging as typically behavior change is a complex process [13]. Even when these practices are supported by evidence-based guidelines and staff training, compliance is not assured, as numerous factors beyond knowledge and skills come into play. These factors include social settings (e.g., social norms), organizational context (e.g., capacity and resources), and psychological and cognitive factors (e.g., attitudes) [14–16].

Behavioral Sciences can serve as a valuable tool for developing theory-based interventions to enhance compliance with IPC measures, such as the proper utilization of PPE [17]. BI involves leveraging behavioral sciences to analyze and address practical issues in real-world settings, often coupled with a rigorous evaluation of the effects of interventions. The term BI describes organizational efforts to apply evidence-based insights into human behavior, informing processes and policies. BI draws from various disciplines, including design thinking, psychology, and behavioral economics, to craft policies and programs that resonate with real people in their specific contexts. BI projects utilize behavioral theories, frameworks, and mixed-methods studies to explore the drivers and facilitators of behavior and to design interventions aimed at fostering positive behavioral change [18–21]. Currently, only a few studies have utilized behavioral theory such as the Theoretical Domains Framework (TDF) to improve IPC practices [17,22]. However, the TDF framework has previously been applied during the COVID-19 pandemic to understand behavioral drivers of IPC practices [23].

In our previous theory-based mixed-methods study, we assessed behavioral factors influencing compliance with COVID-19-specific IPC measures among HCWs in LTCFs during the pandemic [24]. Our study identified many barriers, including a lack of management reinforcement, insufficient staffing, and lack of confidence in the ability to apply IPC measures during the pandemic, suggesting that adherence to PPE was a significant IPC challenge. Recognizing that behavioral barriers and facilitators are context-specific, we subsequently conducted a follow-up study specifically to investigate the use of PPE when caring for COVID-19 residents in LTCFs. Additionally, we assessed the availability of guidelines and training opportunities on PPE use during the pandemic.

## Methods

### Study design

This mixed-methods study employed both a survey, utilizing a convenience sample of HCWs in Finnish LTCFs with 24-hour care services, and a subsequent qualitative study. The Theoretical Domains Framework (TDF), serving as a BI-based theoretical framework, was utilized in both study components to comprehend the behavioral barriers and enablers associated with PPE use. The original TDF integrates commonly related constructs into 12 domains to facilitate the identification of influences on behavior [25]. In our study, we utilized an 18-domain version of the framework [26]. which explores cognitive, affective, social, and environmental influences on behavior. TDF amalgamates elements from various theories and was developed to support evidence-based practice implementation, with a specific focus on effecting behavioral change among health professionals [27–29].

### Survey

We identified relevant studies that had used the TDF framework among healthcare providers and used those as references in developing our questionnaire. the [13,26,30]. We chose to include 15 of these domains in our survey after thorough discussions and prioritization of each construct through a process of elimination. Our goal was to retain the most relevant and observable key construct for each domain within the LTCF context in Finland. However, we made an exception for the “social influences” domain, deciding to include two constructs—subjective norms and social support—in our survey. This choice was made to ensure a more comprehensive understanding of the dynamics within this specific domain.

In developing our questionnaire, we created one statement for each construct, emphasizing questionnaire length to reduce respondent fatigue. These statements were adapted from previous studies using the TDF to understand healthcare professionals’ behaviors [13,26,30]. An exception was made for the “social influences” domain, where two statements covered subjective norms—addressing peer and manager expectations. Additionally, in the “organization” domain, we included two questions on organizational support and resources, assessing supplies and human resources sufficiency. This decision aimed to provide a comprehensive understanding of these aspects within the organizational context.

The statement development process relied on the collective experience of the authors in public health, infection prevention and control, and question development. The statements were phrased both positively and negatively, and respondents were asked to rate their level of agreement with each statement on a four-point Likert scale (1=strongly agree; 2=agree; 3=disagree; 4=strongly disagree; 5=cannot say).

We conducted piloting in three different LTCFs to assess whether the items were worded, had face validity for the constructs being measured, applied to the context, and could be completed within a reasonable time frame. In the second round of piloting, we tested the functionality of the online survey platform hosting the questionnaire by having research team members enter the online questionnaire.

The final questionnaire comprised 18 statements measuring 15 TDF domains and included questions about HCWs and LTCFs’ background information (n=6, including profession, type, owner and resident mobility of the facility, municipality, healthcare district), IPC guidelines for COVID-19 and training (n=5), and PPE non-compliance, specifically difficulties experienced in using PPE by IPC guidelines when caring for residents with COVID-19 (n=2, overall and by type of PPE. The questionnaires were translated from Finnish to Swedish and English. TDF domains, constructs, and related statements can be found in Table 1.

**Table 1:** Theoretical domain framework and related survey statements (n=18).

Domain	Construct	Survey statement
Knowledge	Education	I have received enough training on how to use personal protective equipment.
Skills	Skills	I can use surgical mask following the guidelines for caring for COVID-19 patients.
Beliefs in capabilities	Self-efficacy	I can perform my professional duties and use personal while caring for COVID-19 patients.
Social influences	Subjective norm	My colleagues think that we all should use personal protective equipment when we care for COVID-19 patients.
Social influences	Subjective norms	My bosses think that we all should use personal protective equipment when we care for COVID-19 patients.
Social influences	Social support	Feedback and support from bosses influence how well I follow the guidelines of personal protective equipment when providing care for COVID-19 patients.
Behavioral regulation	Action planning	I have given some thinking about how to use surgical masks when providing care for COVID-19 patients.
Beliefs about consequences	Outcome expectations	Use of personal protective equipment when providing care for COVID-19 patients is beneficial.
Nature of behaviors	Automaticity	The use of personal protective equipment when providing care for COVID-19 patients has become routine to me.
Organization	Organizational support and resources	I have enough personal protective equipment when I provide care for COVID-19 patients.
Organization	Organizational support and resources	We have enough staff members to allow me the time necessary to use personal protective equipment according to the guidelines when caring for COVID-19 patients.
Negative emotions	Negative emotions	The use of personal protective equipment when caring for COVID-19 patients is stressful.
Positive emotions	Positive emotions	Use of personal protective equipment when caring for COVID-19 patients creates a sense of safety.
Goal	Priority	By using personal protective equipment based on the guidelines when caring for COVID-19 patients, I contribute to the control of the pandemic.
Intention	Intention	I have decided to use personal protective equipment as long as the guidelines require.
Patient influences	Patient characteristics	COVID-19 patients consider my usage of personal protective equipment as positive.
Socio-political context	Socio-political context	Health authorities (e.g., municipality, healthcare district) support the use of personal protective equipment during care for COVID-19 patients with their guidelines.
Innovation strategy	Innovation strategy	Bosses have made the use of personal protective equipment easy when providing care for COVID-19 patients in our unit.

COVID-19, coronavirus disease 19.

The survey data were collected using a web-based questionnaire. All HCWs who had provided care for COVID-19 patients in LTCFs were eligible to join the study. Since, there was no central database of all Finnish LTCFs, the recruitment for the survey took place through regional infectious disease physicians (n=20) who were responsible for communicable disease control in the healthcare district. They were asked to share the survey link via email with the management of LTCFs in their healthcare districts who would then invite the HCWs to participate in the survey. The survey was open for two months during May-June 2022. Respondents participated anonymously but they could also provide their contact information to participate in an in-depth interview.

The univariate analysis and multivariate model were based on logistic regression. Model building was challenging due to collinear variables, sparse data, and limited variation in responses. For the analyses, the scale of TDF domains-related questions were therefore recategorized as a binary variable (agree/not agree). We performed a forward selection of variables using Akaike information criteria (AIC) when evaluating the best selection of variables and interactions for the final model. The outcome was responding ‘yes’ to the survey question, experiencing difficulties in complying with PPE guidelines, and variables were based on replies to TDF domains-related questions (n=18) as well as the background information of the HCWs (profession) and LTCFs (type, owner, and resident mobility) and training (n=2). Statistical analysis was performed using Stata version 17.0 (StataCorp LLC, TX, USA).

### Qualitative study

In our qualitative study, we aimed to get a better understanding of the TDF domains that were identified in the survey as statistically significant barriers to health worker compliance with PPEs. We conducted in-depth interviews by phone based on a question guide developed after completing our survey analysis. Our guide covered IPC guidelines training and TDF domains that were statistically associated with PPE compliance in the survey to get a better understanding of them. We asked open-ended and semi-structured questions, supplemented with probes to facilitate discussion.

Respondents from the survey who expressed interest in participating were contacted by our team members (ALL) for interview scheduling. We made two additional contact attempts, one by email and another by phone. The researcher (ALL), who is trained to collect qualitative data and analysis methods, conducted the interviews in June 2022 and August 2022. All interviews, lasting approximately 60 minutes, were audio-recorded, and the participant’s verbal consent was audio-recorded before the start of each interview. Confidentiality was maintained, and no recorded identifiers were used. Professional transcribers transcribed the audio data.

The analysis was based on a framework analysis that followed the TDF domains so that inductive coding was conducted in each domain separately followed by exploring possible connections and overlaps between the domains. The process started with a data familiarization during which the analyst read all the transcripts a few times to get an overall idea of the dataset and to create an initial set of codes. During the coding, emerging new codes were included, followed by refining, expanding codes, and developing categories [31]. NVIVO12 was used in the coding process. The initial analysis was shared with the study team (JML, OL) to get a consensus on the emerging categories. In the final stage, the analyst (ALL) developed the interpretation. The synthesis of the results served as the foundation for operational recommendations to improve HCWs’ compliance with the use of PPE when providing care for residents in LTCFs,

### Ethics approval and consent to participate

The study was approved by the Helsinki University Hospitals Ethical Review Board in September 2020 (HUS/2434/2020). All respondents participating in the qualitative interviews gave verbal consent for their participation and recording of the interview.

## Results

### Survey responses

A total of 373 HCWs (median 31, range by healthcare districts, 1–83) from 11/20 healthcare districts responded to the survey, 369 in Finnish and four in Swedish. As many as 86.3% of the respondents worked in 24-hour service housing for the elderly (322/373) and 72.3% in the public sector (266/368). Of the respondents, 60.6% were assistant nurses (226/373) and 32.2% were registered nurses (120/373) (Table 2).

**Table 2:** Background information of 373 survey participants working in long-term care facilities, Finland, May-June 2022.

Variable	N	%
**Profession**		
Assistant nurse	226	60.6
Nurse	120	32.2
Physician	0	0
Other *	27	7.2
**Type of facility**		
Sheltered housing with 24/7 care services	322	86.3
Nursing home/healthcare center inpatient ward	51	13.7
**Owner of the facility**		
Public	266	72.3
Private	102	27.7
**Estimated proportion of residents moving without assistance outside their own room in the facility**		
>50%	173	46.4
<50%	163	43.7

^a^managers, social workers, nursing aides, and students

Overall, 99.5% of the respondents (368/370) reported that there were IPC guidelines for COVID-19 in their LTCF. We had 55.8% of the respondents (207/371) reporting having received training related to the IPC guidelines. From the sample, 47.4% had been trained by the healthcare district or municipality (111/234), and 18.8% by a staff member in their organization (44/234) or 22.658% by unit (53/234). From the sample, 11.1% of the respondents (26/234) reported other training sources, such as self-learning (8/26), supervisors, managers, and colleagues (9/26), or previous jobs or schools (3/26). From our respondents 28.5% received training online (65/228), 25.4% face-to-face (58/228), and 22.4% by watching a pre-recorded video (51/228). Other training tools used by 23.7% of respondents (54/228) included written and online materials (31/54) and short, informal learning sessions (14/54); the remaining respondents did not specify their training (8/54). Of the respondents, 70,4% (159/226) reported having had an opportunity to ask questions during their training. Of the respondents, 70.2% (262/373) reported that they had experienced difficulties in complying with PPE guidelines when caring for residents with COVID-19. As much as 67.9% of the respondents reported difficulties when using FFP2 and FF3 respirators (178/262), followed by 56.1% with visors (147/262), 46.9% with gowns (123/262), 30.9% with goggles (81/262) and 27.5 with surgical masks (72/262). The univariate analyses identified three TDF domains significantly associated with experiencing difficulties in complying with PPE guidelines when caring for COVID-19 patients included (skills, organization, and negative emotions, Table 3), and in the final model only two of them (organization and negative emotions, Table 4). The first was organization, which referred to sufficient human resources being a facilitator to comply with PPE guidelines. The second was negative emotions, which referred to stressful feelings and as a barrier to complying with PPE guidelines.

**Table 3:** Theoretical domain framework, related survey statements and their associations with compliance in personal protective equipment use among healthcare workers in long-term care facilities, univariate logistic regression analysis, Finland, May-June 2022 (n=371).

		Variable		Logistic regression		
Domain	Construct	Survey statement (n=18)	% (agreed/responded, n/N)	OR	95%CI	p value
Knowledge	Education	I have received enough training on how to use personal protective equipment.	82.4 (305/370)	0.97	0.41-2.31	0.95
Skills	Skills	I can use surgical mask following the guidelines for caring for COVID-19 patients.	97.8 (362/370)	2.23	1.15-4.32	0.02
Beliefs in capabilities	Self-efficacy	I can perform my professional duties and use personal while caring for COVID-19 patients.	88.1 (325/369)	0.35	0.08-1.48	0.10
Social influences	Subjective norm	My colleagues think that we all should use personal protective equipment when we care for COVID-19 patients.	90.6 (336/371)	1.88	0.67-5.27	0.26
Social influences	Subjective norms	My bosses think that we all should use personal protective equipment when we care for COVID-19 patients.	98.6 (362/367)	1.15	0.33-4.05	0.83
Social influences	Social support	Feedback and support from bosses influence how well I follow the guidelines of personal protective equipment when providing care for COVID-19 patients.	72.7 (266/366)	1.29	0.60-2.79	0.52
Behavioral regulation	Action planning	I have given some thinking about how to use surgical masks when providing care for COVID-19 patients.	93.5 (345/369)	1.23	0.27-5.68	0.79
Beliefs about consequences	Outcome expectations	Use of personal protective equipment when providing care for COVID-19 patients is beneficial.	94.9 (351/370)	1.40	0.30-6.57	0.68
Nature of behaviors	Automaticity	The use of personal protective equipment when providing care for COVID-19 patients has become routine to me.	90.0 (332/369)	2.03	0.78-5.29	0.17
Organization	Organizational support and resources	I have enough personal protective equipment when I provide care for COVID-19 patients.	86.0 (318/370)	0.70	0.24-2.07	0.50
Organization	Organizational support and resources	We have enough staff members to allow me the time necessary to use personal protective equipment according to the guidelines when caring for COVID-19 patients.	54.9 (202/368)	0.47	0.22-0.96	<0.05
Negative emotions	Negative emotions	The use of personal protective equipment when caring for COVID-19 patients is stressful.	77.8 (288/370)	2.22	1.10-4.50	<0.05
Positive emotions	Positive emotions	Use of personal protective equipment when caring for COVID-19 patients creates a sense of safety.	84.4 (313/371)	0.88	0.33-2.35	0.79
Goal	Priority	By using personal protective equipment based on the guidelines when caring for COVID-19 patients, I contribute to the control of the pandemic.	89.7 (332/370)	1.65	0.59-4.59	0.36
Intention	Intention	I have decided to use personal protective equipment as long as the guidelines require.	96.8 (358/370)	3.05	0.78-11.97	0.14
Patient influences	Patient characteristics	COVID-19 patients consider my usage of personal protective equipment as positive.	49.3 (181/367)	1.20	0.55-2.58	0.65
Socio-political context	Socio-political context	Health authorities (e.g., municipality, healthcare district) support the use of personal protective equipment during care for COVID-19 patients with their guidelines.	83.9 (308/367)	0.45	0.10-1.97	0.24
Innovation strategy	Innovation strategy	Bosses have made the use of personal protective equipment easy when providing care for COVID-19 patients in our unit.	79.6 (293/368)	0.43	0.13-1.45	0.13

COVID-19, coronavirus disease 19; OR odds ratio; CI, confidence interval.

**Table 4:** Multivariate model: theoretical domain framework, related survey statements and their associations with compliance in the use of personal protective equipment among healthcare workers, in long-term care facilities, Finland, May-June 2022 (n=310).

Variable				
Domain	Survey statement	OR	95%CI	p-value
Organization	We have enough staff members to allow me the time necessary to use personal protective equipment according to the guidelines when caring for COVID-19 patients.	0.34	0.18-0.63	<0.01
Negative emotions	The use of personal protective equipment when caring for COVID-19 patients is stressful.	2.92	1.51-5.67	<0.01
**Background information**				
Worker group (nursing assistant)		1.54	0.88-2.71	0.13

COVID-19, coronavirus disease 19; OR, odds ratio; CI, confidence interval.

### Qualitative study

Of the total 373 HCWs, 49 survey respondents initially agreed to schedule an interview. However, by the time the interviews were scheduled only 22/49 were available for an interview because eight changed their mind, five were no longer working in the LTCF, and 19 did not respond despite the three contact attempts. Interviewees included 14 assistant nurses and eight registered nurses. All were female, most of them (17/22) had more than 10 years of working experience, and over half (13/22) worked in the private sector.

### Training and education

Many interviewees explained that they only received some informal, on-the-job guidance, which they did not consider as training. Several interviewees explained that lack of training made them feel neglected as at the same time they were under enormous pressure not to spread the virus. Several interviewees also described their informal sessions to learn about IPC procedures as short because they were often linked with morning meetings that had many other items on the agenda. A couple of interviewees clarified that they had a training session at the beginning of the pandemic but by the time they needed to use PPE, they had forgotten how to do it. Several interviewees also explained that they did not receive any training, but instead, their peers explained what to do. Some interviewees explained that they only had received written instructions with pictures of how to put on and take off PPE, which did not make them confident using them. One respondent said that she understood only very late during the pandemic how putting on and taking off PPE is critical.

Some interviewees, however, clarified that they had received several training sessions including an opportunity to practice putting on and taking off PPE. Many of these interviewees explained that the IPC nurse from the municipality had trained them. Some highlighted that the training included demonstrations or practice on how to put on and take off PPE. Some noted that they had received links to video recordings that were helpful reminders. One interviewee, who was also an IPC trainer, pointed out that she did refresher training continuously as the use of PPE was something new for the staff and because there was often time between the outbreaks, during which staff forgot the procedures.

*“I had no idea that it matters how we put on and take off PPE. This was the first time I was using them. It was good that they made us take the training sessions every now and then.” (Assistant nurse, private facility)*

### Factors associated with PPE compliance and coping mechanism

Interviewees were asked to describe further how the lack of sufficient human resources (TDF Domain: Environmental Factors) and negative emotions (TDF Domain: Emotions) influenced negatively the update of PPE(barriers) and what kind of coping mechanisms (enables) they had developed. Table 5 shows the themes of the analysis.

**Table 5:** Qualitative analysis: theoretical domain framework and related themes associated with compliance in the use of personal protective equipment and coping mechanisms.

	Theme	Sub-theme
Environmental factors: human resources	1: Lack of time	
	2: Adaptation	
	3: Team support	
Emotions: negative emotions	4: Stress	4.1 a reminder about the crisis 4.2 Physical symptoms 4.3 Logistical challenges
Coping Mechanisms	5. Self-reminders of the importance of PPE use 6. Proactively searching for better suited PPE	

### Domain: Environmental factors: human resources

#### Theme 1: Lack of time.

Most interviewees clarified that insufficient staffing in their workplace gave them less time to follow IPC procedures such as proper use of PPEs.

*“It is all about the time. If we have enough staff, we have time to follow guidelines, socialize and take care of the residents. “(Assistant nurse, private facility)*

Many interviewees clarified that putting on and taking PPE off was time-consuming as patient care required repeating the procedures several times daily. Some interviewees explained that when they had several residents with COVID-19 which required that they put on and take off PPE more than 30 times per work shift.

*“Using the PPEs made me late for my visits to the residents. I felt sorry that they had to stay so much alone in the room, but the use of PPE took from me so much time that I just couldn’t keep up.” (Assistant nurse. Private facility)*

#### Theme 2: Adaptation.

Interviewees explained that the ability to adapt to the pandemic situation with new IPC procedures facilitated compliance.

*“You cannot continue just as business usually but to look for ways to manage the situation.” (Assistant nurse, public facility)*

Some interviewees explained that they used modified procedures to manage their tasks and use PPE. For example, interviewees said that they did not always change their masks when going from one COVID-19 patient to another, but they made sure to wash their hands and change their gloves. Some interviewees clarified that when the ward had more than five residents with COVID-19, they provided care for all patients in the same space without changing PPE in between. Many interviewees mentioned having merged several tasks into one visit in the patient room, which reduced the number of visits they made to the patient per working shift.

*“Yes, we had to be innovative and find new ways to do our job because the use of PPE took so much time. For example, during the mid-day visit, we also did things that we would normally do in the afternoon that allowed us to skip one entire visit to the patient room.” (Assistant nurse, public facility)*

#### Theme 3: Teamwork.

Many interviews also highlighted that teamwork allowed them to manage duties including the use of PPEs.

*“If your workplace is a community that works together, then the work even in such a crisis as the pandemic can run better. But all facilities have not managed to build this type of community. (Nurse, private sector)*

Many interviewees also referred to team spirit and the support of colleagues to help one another as an important coping mechanism to comply with the use of PPE when caring for residents with COVID-19. For example, one interviewee explained that while she was with the patient, her colleagues would take the patient’s lunch tray outside of the room so that she did not have to leave the room and take off her PPE.

*“I think I had not quite managed to do what I was asked to do when I had residents with COVID-19. So many times, putting on and taking off PPE… But I had my colleagues who helped me morally by cheering me up but also helping me if I forgot something. For example, they brought it [the lunch tray] outside the room.” (Assistant nurse, private facility)*

### Domain: Emotions- negative emotions

#### Theme 4: Stress.

The participants were asked to explain how negative emotions influenced their use of PPEs. Many participants reported experiencing stress because the demands to use PPE, amplified their emotional and physical burden.

*“I cannot focus because using PPE and worrying about whether I am using it correctly is always on my mind.” (Assistant Nurse, Private Facility)*

##### Sub-theme 4.1: A reminder about the crisis

Many interviewees highlighted that the use of PPE was a constant reminder that they were in crisis and the pandemic was ongoing. Some interviewees explained that their mood changed immediately when they put on PPE.

*“When I wear PPE, I feel that the pandemic is real, and we are in the middle of it. It used to make me feel so hopeless. I would rather forget that I am in the pandemic but the mask and the gloves and all other gear do not allow me to do so.” (Assistant nurse, public facility)*

##### Sub-theme 4.2: Physical symptoms

Many interviewees also explained that PPE-related stress also developed from the physical symptoms of using masks. Several interviewees explained that wearing masks for long periods gave them headaches and made them tired. Some respondents also referred to allergic skin reactions due to mask use. Some interviewees highlighted that the use of an apron made them sweat.

*“Most of the time I feel either wet from the sweat or hot. Sometimes I feel that I cannot breathe. So, when using PPE, yes, I am stressed out.” (Nurse, public facility)*

##### Sub-theme 4.3: Logistical challenges

Some interviewees clarified that the use of PPE presented practical challenges. They were stored far away, and a dedicated place to put on and take off PPE was either missing or in the middle of a busy facility where it was not possible to concentrate on the correct procedures. Some interviewees said that while putting on or taking off PPE, they often came in contact with COVID-19-negative residents, which caused stress as nobody wanted to be blamed for transmitting the virus within the facility. Some interviewees explained that the PPE disposal area was often far across the facility, which they perceived as a risk factor and another reason for stress.

*“There are just so many practical issues that make us stressed out. The place where we put on and take of PPE usually changes based on where the COVID-19-positive residents are located. So, every time you have to think about these practical issues again. The problem is that this is not a hospital.” (Assistant nurse, private facility)*

### Coping mechanisms to manage stress

Interviewees were asked how they minimized stress related to the use of PPE.

#### Theme 5: Self-reminders of the importance of PPE use.

Some interviewees explained that they remind themselves that using PPE is something very important as they all follow the same procedures nationwide.

##### “I thought this must be important since we all have to use PPE.” (Nurse, public facility).

#### Theme 6: Proactively searching for better-suited PPE.

Some interviewees explained that they were actively looking for more suitable masks that would be easier to use and discussing them with management and colleagues.

*“We got some masks that are easier to use after we spoke to our supervisor. They care about us. I had much less stress at work when I could breathe better.” (Assistant nurse, private facility)*

## Discussion

Our mixed-methods study provided useful insights into the utilization of PPE in LTCFs. To gain a more comprehensive understanding of the factors influencing PPE compliance, we applied a theoretically informed framework, TDF. Our analysis revealed one significant behavioral barrier – stress induced by PPE use – and one behavioral facilitator – the presence of adequate nursing staff – influencing the utilization of PPEs. Although PPE-related guidelines were readily available within the facilities, a noteworthy observation is that a substantial number of staff members did not receive any training on how to implement these guidelines.

Our survey aimed to identify factors influencing the use of PPE, and our interviews focused on understanding how these factors influenced the PPE use strategies for nursing staff to overcome these barriers. Our findings underscored that insufficient nursing staff posed a significant obstacle, particularly due to the time-consuming nature of PPE use when caring for multiple residents. Interviewees shared various coping and time management methods, such as grouping tasks during patient visits to minimize the number of required visits. This aligns with the guidance from the European Centre for Disease Prevention and Control (ECDC), recommending the minimization of personal contacts and the adaptation of activities within healthcare facilities [9]. Additionally, interviewees reported practices inconsistent with IPC guidelines, such as not changing PPE when visiting COVID-19-positive residents—a phenomenon previously observed in other regions due to PPE shortages [32]. Notably, there were no PPE shortages in Finland at the time of our study.

Our research revealed that the appropriate use of PPE faced challenges due to negative emotions, especially stress. This stress was linked to constant reminders of the crisis, physical discomfort, and logistical challenges associated with PPE use. These findings align with a study conducted in the UK during the pandemic, where healthcare workers reported stress and anxiety due to logistical challenges and physical symptoms such as overheating [33].

While pandemic-related fatigue among nurses has been extensively studied, the specific topic of PPE-related stress during the pandemic remains relatively unexplored [34–36]. Emotions play a crucial role in shaping risk perceptions and subsequent behaviors [37]. Moreover, negative emotions can override logical reasoning based on numeric information and probabilities, complicating risk communication [38]. Negative emotions also tend to bias decision-making towards negative information, underscoring the importance of addressing these emotional aspects [39]. A study conducted in New Zealand during the pandemic explored healthcare workers’ experiences with PPE use, highlighting values such as transparency, trust, safety, and respect [40]. Personal values can significantly influence emotional responses, particularly when behaviors or circumstances conflict with what individuals hold as important. A deeper understanding of values could provide valuable insights into the negative emotions associated with PPE use, as reported by respondents in our study.

To address these behavioral barriers, we can employ a theoretically informed framework such as the Behaviour Change Wheel (BCW). The BCW breaks down TDF categories into relevant intervention functions and policy categories [19]. Our study methodology is aligned with a recent study in England that likewise used TDF to identify behavioral barriers to PPE use and social distancing among healthcare workers and BWC to develop behaviorally informed interventions [41]. In our study, the issue of insufficient human resources falls under the TDF category of environmental context and resources, corresponding to intervention functions like incentivization and coercion and policy categories related to environmental and social planning in the BCW. For instance, a BCW-informed policy decision could involve appointing a designated person, such as a link nurse, within each LTCF. This individual would lead and support the implementation of IPC measures, supported by LTCF managers. Training these link nurses could be conducted by regional IPC experts, who can also provide consultation during outbreaks and guide the implementation of control measures.

Likewise, the BCW can inform appropriate behavior change interventions when addressing the emotional domain of the TDF, aligning with BCW intervention functions like enablement and training, aiming to reduce barriers and enhance capability [8]. In this context, stress management programs accessible within the workplace could serve as suitable interventions. Psychology has developed tools and models to improve emotional experiences and socio-emotional behavior. Practical interventions and training tested in various settings [42,43] offer the potential to mitigate HCWs’ negative emotions associated with PPE use. Recent reviews emphasize the significance of individual-level interventions and self-care strategies in stress reduction, highlighting the effectiveness of technology paired with cognitive-behavioral components [44]. For instance, a virtual reality protocol designed to address quarantine-related stress could be adapted to manage stress related to PPE use [45]. Additionally, interventions focusing on self-assessment of stress levels have shown promise in reducing stress [46,47].

The availability of guidelines and the possession of adequate knowledge and skills to adhere to these guidelines are essential prerequisites for the proper utilization of PPE [22,48]. In our study, it was evident that many HCWs had not undergone training on PPE use, emphasizing the critical importance of universal access to high-quality training. A study conducted in the UK concluded that numerous uncertainties related to IPC in LTCFs could have been addressed with timely, fact-based guidance [49].

Our findings also revealed that some LTCFs relied solely on written instructions or brief informal guidance from managers or peers regarding the proper procedures for donning or doffing PPE. Additionally, IPC training was not consistently provided by IPC professionals. Studies conducted elsewhere underscore the significance of specialist support for IPC training as a crucial component of effective COVID-19 outbreak management in LTCFs globally [50,51]. The identified gaps in IPC training underscore the necessity of considering a standardized training curriculum for PPE use during outbreaks, particularly in decentralized health systems like Finland. One potential solution is to enhance the PPE training curriculum in nursing schools to encompass standard precautions and the transmission routes of microbes, in addition to fundamental knowledge. Notably, our study highlighted that many LTCF trainings during the pandemic were conducted online due to restrictions on face-to-face meetings. This underscores the need to reassess the organization of PPE-related training during crises in the future.

Research indicates that online video training on PPE use can enhance knowledge about COVID-19 protection, and improve learning achievement, critical thinking skills, and learning self-efficacy [51]. The development of national training materials on putting on and removing PPE could potentially alleviate the workload of local and regional IPC experts. However, it is crucial to account for different contexts and the availability of various types of PPE in such initiatives.

A few limitations should be considered when interpreting our study findings. Firstly, we only covered three TDF domains thoroughly in the interviews since it was feasible to carry out long interviews with healthcare workers to cover all domains. Therefore, we conducted a survey, and based on the survey results three TDF domains were selected for the interviews. Secondly, selection bias is possible, as survey participants and interviewees were likely more interested and motivated in using PPE. While we were able to assess the representativeness of survey respondents across regions (encompassing more than half of the regions), there were no male participants. Moreover, the survey was conducted in Finnish or Swedish (none in English), potentially excluding nursing assistants with foreign backgrounds. Studies have indicated a higher COVID-19 risk among male HCWs and nursing assistants with foreign backgrounds [52], warranting specific attention in training and implementing infection control measures. Lastly, social desirability bias might have influenced our qualitative findings, considering the sensitivity surrounding COVID-19 outbreaks in LTCFs. Nonetheless, a notable strength of our study lies in utilizing a behavioral framework to identify key variables associated with PPE compliance, facilitating the development of theory-based behavior change interventions [27].

## Conclusions

Our study may guide preparedness training for future epidemics by providing behaviourally informed recommendations on how to improve compliance with PPE use. These include ensuring universal access to standardized training on the use of PPE, carrying out interventions that address negative emotions and stress related to the use of PPE, and ensuring adequate human resources that match the changing work responsibilities during epidemics.
